# Are museums the future of evolutionary medicine?

**DOI:** 10.3389/fgene.2022.1043702

**Published:** 2022-11-11

**Authors:** Philippe Charlier, Virginie Bourdin, Anaïs Augias, Luc Brun, Jean-Blaise Kenmogne, Erol Josue

**Affiliations:** ^1^ Department of Research and High Education, Quai Branly—Jacques Chirac Museum, rue de l’université, Paris, France; ^2^ Laboratory Anthropology, Archaeology, Biology (LAAB), Paris-Saclay University (UVSQ), Paris, France; ^3^ Foundation Anthropology, Archaeology, Biology (FAAB), Institut de France, Paris, France; ^4^ Department of Pathology, University Hospital, Parakou, Benin; ^5^ CIPCRE, Bafoussam, Cameroon; ^6^ National Bureau of Ethnology, Port-au-prince, Haïti

**Keywords:** paleogenetics, paleomicrobiome, evolutionary medicine, paleopathology, biomolecular engineering

Evolutionary medicine (or Darwinian medicine) is considered a branch of medicine and modern biology seeking to understand the mechanisms causing the onset of diseases and their evolution (and intricacy) over time. By examining the evolutionary processes of the human species, infectious agents, carcinogenic factors and the environment, it is thus possible to identify evolutionary trends, ruptures, and interactions leading to therapeutic escapes, resistance to antibiotics, and carcinogenesis or autoimmunity phenomena (Gluckman et al., 2009).

Can museum objects replace or complement “classic” biological samples (i.e. DNA from living humans/animals) for population genetics and evolutionary medicine studies? This perspective could respond to certain ethical reflections, and in particular those concerning the scientific over-solicitation of the populations studied and the impacts generated on their life and functioning—impacts that have already tainted the relationship between scientists and populations. Faced with the distrust of certain Indigenous peoples regarding the fear of the theft of their genetic (and globally bio-cultural) heritage, is it possible to draw legally and respectfully from these objects constituting the collections of museums of ethnology, ethnography, anthropology and natural history?

We have recently been able to show the interest of a bio-molecular study of the typing of lice subspecies on a set of six reduced heads (tsantsas) from the Achuar/Jivaro (Bolivia/Ecuador): beyond the precise identification of ectoparasites, it also highlights that historical migratory movements can be reconstructed (in particular clades that were carried to America by an ancestral Eurasian Beringian population thousands of years ago) (Amanzougaghene et al., 2022).

Other scientific work is possible, which would provide information as much about the physical reality of the object as about its journey and/or the history of the population from which it came. Currently, at the quai Branly–Jacques Chirac museum (Paris, France), we are developing the bio-molecular analysis of partially carbonized residues in a dozen African pipe mouthpieces and chambers (sub-Saharan Africa, Central Africa, and East Africa): if the proteins of botanical species could be highlighted (paleo-proteomics), allowing the identification of the type of plant smoked (tobacco, cannabis, other local species), human DNA related to the saliva of the smoker(s) will most certainly also be amplified in parallel (Schablitsky et al., 2019) providing direct data dealing with the owners and users of such artifacts.

The same is true for our upcoming work on Papua New Guinea penis sheath (koteka) for which genetic analysis should aim to identify bacterial, viral, parasitic, and mycotic species related to the uro-genital and cutaneous microbiome of this male population. In reality, other elements could also be identified, starting with pathogenic species, but also the male and female DNA of the penis sheath wearer and/or the sexual partner(s).

Our team is about to begin the molecular study of the constituents of a wall from the tomb of a king of Abomey (Benin) dated from the end of the 19th century, in order to test for the presence of human blood proteins: initial examination and further exploitation of the results are bound to require the prior authorization of members of the political (current king of Abomey) and spiritual (voodoo clergy) communities in addition to the cultural and heritage authorities, as well as national authorities (ministry of culture, ministry of research, ministry of autochthonous affairs, *etc.*). The same with artefacts conserved in museum and considered, for some, as « desacralised » (for example the ancient throne of the 19th c. king Ghezo associated with four human dried skulls so-called « defeated victims of the Oyo people », conserved in the National Abomey museum, Benin).

For sub-Saharan Africa, many non-desacralised or partially desacralised fetishes still bear significant crusts or drippings of sacrificial materials on their surface, and sometimes in their internal ducts (Mazel and Richardin, 2006; Charlier et al., 2020); their analysis showed the frequent presence of palm oil, flour, and animal blood, but anthropology teaches us that human projections can also be present (sputum, hair, blood - sometimes menstrual -, semen, excrement, placenta, *etc.*) in contexts as diverse as a boli fetish (Mali), an ancestor Bamileke “talking skull” (Cameroon) or a Legba altar (Benin or Togo). These types of surface deposits are not limited to the African continent: in the context of Tantric Buddhism, sections of the human skullcap (kapala) present frequent internal deposits corresponding to a mixture of alcohol, blood, milk, and sperm. The same is true for Hindu lingam and yoni, with organic residues often being associated with these worshipping materials (even from an archaeological origin, due to a partial mineralisation at the surface of the stone).

What about more standard samples like hair, teeth, skulls, and bones, for which genetic exploitation seems more logical or usual? Beyond being simple museum objects, these human remains, either intact or transformed (reduced head, relic, over-modeled skull, scalp, *etc.*) are all biological material readily available to the bio-molecular researcher. Archaeological excavations (and their preserved artifacts) thus become a legitimate and original training ground for specialties that were not initially obvious: biology, medicine, genetics, organic and mineral chemistry. The educational potential of the human and fundamental sciences is demonstrated in each case study published in the biomedical literature, making it possible to reconstruct such aspect or detail of past populations, and their evolutionary phenomena.

Due to their globalizing vocation and openness to non-European cultures, anthropological, ethnographic, and natural history museum are rich in human remains integrated into pieces of art: the skulls surrounding a Bamoun royal calabash (Cameroon, Grassland) are another example for which it would be interesting to seek a relationship between the different individuals (in which case these skulls would be those of protective ancestors against any poison present within the liquid conserved into the calabash, just like a magical antidote); another hypothesis would be that of defeated enemies, whose genetic map would not correspond to that of ancient or modern Bamoun populations, and who would not present any family ties. Present on many dental remains (including those of this object from sub-Saharan Africa), dental calculus is another target of paleogenomics (Warinner et al., 2014): it contains and preserves the DNA of the oral microbiome, to be related with possible oral and/or systemic pathologies, or environmental disturbance (Charlier et al., 2019); sequencing these viral/bacterial/fungi/parasite genomes also contributes to phylogenetic tracing by comparing existing databases (Huynh et al., 2016).

It is important to note that the new perspectives opened up by proteomics, molecular biology and paleogenetics all have analytical limits imposed by the conservation conditions of the source museum objects. Even though remaining macroscopically stable, they undergo degradation due to changes in temperature, pH, humidity, UV-rays exposure, and/or salinity (Pruvost et al., 2007). The contamination of ancient DNA by exogenous modern DNA is a feared pitfall during PCR amplification: in addition to its pre-museum life, the exhibited object undergoes manipulations (exhibition, restoration, *etc.*) that can corrupt the genetic information it is able to provide. It is of course necessary to underline the extreme fragility of the supports studied, and the almost illusory nature of the recommended measures (constant temperature and hygrometry, darkness, wearing of gloves, *etc.*): at the moment of death, each individual’s DNA sequences begin to degrade with some of the constituent nucleotide bases becoming chemically modified (Geigl and Grange, 2014).

While all these studies provide ample opportunities to access crucial information in terms of evolutionary medicine and population genetics, the essential question of data ownership, and the legality of these examinations, still remain. Can museum researchers, who are responsible for preserving and studying the collections they house, still do whatever they want with such objects? Do the same ethical questions apply when Indigenous communities no longer have a living representative or descendant, or for paleontological or archaeological samples where *a priori* links to living persons typically do not exist?

Museum institutions, as holders of collections containing biological resources, are subject to a legal framework that governs access to and use of these resources. Since the adoption of the Convention on Biological Diversity (CBD) in 1992, States have sovereignty over the biological resources within their territory, and the power to determine access to them relies on national governments and legislation. Thus, any access to genetic resources (in a very broad sense, even including even products of metabolism) requires a formal agreement from the provider country (a Mutually Agreed Term must be formalized). It is important to note that it does not matter when the material was collected: any new use is subject to the same principle (see, for example, the French law no. 2016–1087 of 8 August 2016 for the recovery of biodiversity, nature and landscapes). When these resources are associated with traditional knowledge of Indigenous and local communities, which pertains to the majority if not the totality of preserved in ethnographic museums, their prior informed consent is also required, and a sharing benefits mechanism must be negotiated with the provider community (see in this sense the Nagoya Protocol on Access and Benefit-sharing, in force since 2014). Perhaps this also represents an opportunity to re-establish links with the nations concerned by developing mutually beneficial research practices and outcomes that help reduce scientific inequalities. This is without a doubt an opportunity not to be missed for these two communities (indigenous and scientific), and a challenge for the next century to live and work together, each for one another, with total respect for beliefs, properties, practices and customs.

Museums are both managers and holders of biological resource collections, and have an obligation to ensure the respect of these ethical norms. Moreover, museum collections could be considered as a powerful way to promote capacity and capability building to reduce scientific inequalities between nations that curate collections, and nations from where the artefacts originally come from (Fox and Hawks, 2019; Argüelles et al., 2022).
